# Immune Checkpoint Proteins, Metabolism and Adhesion Molecules: Overlooked Determinants of CAR T-Cell Migration?

**DOI:** 10.3390/cells11111854

**Published:** 2022-06-06

**Authors:** Luca Simula, Emma Ollivier, Philippe Icard, Emmanuel Donnadieu

**Affiliations:** 1 Equipe Labellisée Ligue Contre le Cancer, CNRS, INSERM, Institut Cochin, Université Paris Cité, F-75014 Paris, France; luca.simula@inserm.fr (L.S.); emma.ollivier@inserm.fr (E.O.); 2 Service de Chirurgie Thoracique, Hôpital Cochin, Hôpitaux Universitaires Paris Centre, APHP, Paris-Descartes University, F-75014 Paris, France; philippe.icard@aphp.fr; 3 UNICAEN, INSERM U1086 Interdisciplinary Research Unit for Cancer Prevention and Treatment, Normandie Université, F-14000 Caen, France

**Keywords:** T cells, chimeric antigen receptor, migration, PD-1, metabolism, adhesion

## Abstract

Adoptive transfer of T cells genetically engineered to express chimeric antigen receptors (CAR) has demonstrated striking efficacy for the treatment of several hematological malignancies, including B-cell lymphoma, leukemia, and multiple myeloma. However, many patients still do not respond to this therapy or eventually relapse after an initial remission. In most solid tumors for which CAR T-cell therapy has been tested, efficacy has been very limited. In this context, it is of paramount importance to understand the mechanisms of tumor resistance to CAR T cells. Possible factors contributing to such resistance have been identified, including inherent CAR T-cell dysfunction, the presence of an immunosuppressive tumor microenvironment, and tumor-intrinsic factors. To control tumor growth, CAR T cells have to migrate actively enabling a productive conjugate with their targets. To date, many cells and factors contained within the tumor microenvironment have been reported to negatively control the migration of T cells and their ability to reach cancer cells. Recent evidence suggests that additional determinants, such as immune checkpoint proteins, cellular metabolism, and adhesion molecules, may modulate the motility of CAR T cells in tumors. Here, we review the potential impact of these determinants on CAR T-cell motility, and we discuss possible strategies to restore intratumoral T-cell migration with a special emphasis on approaches targeting these determinants.

## 1. Introduction

Adoptive transfer of T cells genetically engineered to express chimeric antigen receptors (CAR) has demonstrated striking efficacy for the treatment of several B-cell malignancies. CAR are synthetic receptors that consist of an MHC-independent antigen binding domain usually derived from a tumor-specific monoclonal antibody fused to an intracellular signaling region, composed of the CD3ζ chain and costimulatory molecules from CD28 and 4-1BB. As of September 2021, five CAR T products have been approved by the Food and Drug Administration (FDA) in the United States, targeting leukemia, lymphoma, and multiple myeloma (for a review see [1]).

However, the success of the CAR T-cell therapy is tainted by the occurrence of relapses and the lack of favorable clinical responses in solid tumors [2]. It is therefore of paramount importance to understand the mechanisms of tumor resistance to CAR T cells. Possible factors contributing to such resistance have been reported, including inherent CAR T cell dysfunction, the presence of an immunosuppressive tumor environment, and tumor-intrinsic factors (for a review, see [3]). To perform their tasks and control the growth of solid tumors, CAR T cells need to migrate actively. It starts with the entry of engineered T cells into the malignant site. Inside the tumor, T cells need to navigate within the stroma, a complex environment that surrounds tumor islets. Then, once they reach tumor islets, T cells have to form productive interactions with cancer cells associated with the delivery of cytotoxic molecules that kill cancer cells. Evidence suggests that several of these migratory steps are defective in solid tumors and thus might contribute to explain the failure of CAR T cells in human carcinomas (for a review see [4]). The reasons for such defective migration remain incompletely understood. A dense extracellular matrix together with a lack of chemoattractant molecules has been demonstrated in some tumors [5]. The objective of this review is to focus on other and overlooked determinants that are susceptible to restrain the trafficking of CAR T cells in solid tumors and therefore prevent them from forming conjugates with their targets. These include the expression of immune checkpoint proteins, the presence of metabolic factors, and the lack of adhesion molecules. We also propose strategies that can be applied to target these determinants to restore efficient migration of engineered T cells and thus their antitumor activities.

## 2. Migration Is Crucial for CAR T-Cell Anti-Tumoral Activities

**Figure 1:**
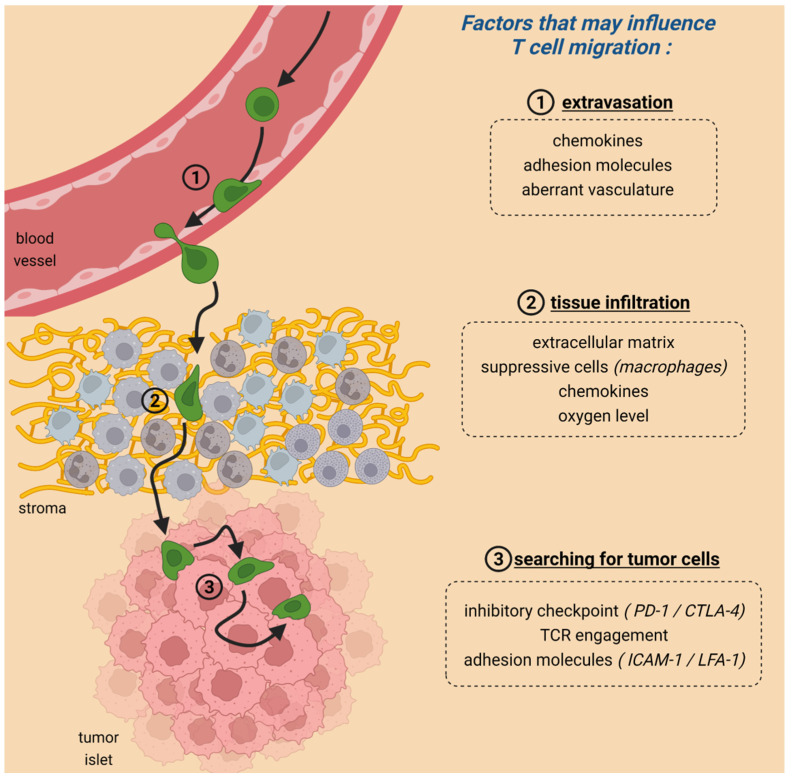
The different steps of T-cell migration in tumors. (1) During the first step, T cells enter the malignant site. (2) Then T cells migrate within the stroma (composed of fibers, in yellow, and several cell types) and (3) finally make contact with tumor cells. All these steps are controlled by a number of cells and components, some of them listed in the figure. Figure created with BioRender.

To achieve tumor regression, CAR T cells need to form productive conjugates with their targets. This cell-cell interaction is the end result of a series of steps during which T cells actively rely on migration (Figure 1). First, T cells have to enter the tumor by crossing the blood endothelial cells. This extravasation step is dependent on chemokines, and adhesion molecules and shows some analogies with the entry of T cells into inflamed peripheral tissues [6]. 

Of note, defects in the entry phase have been reported for non-modified T cells contributing to explaining why some tumors are devoid of T lymphocytes and thus are refractory to immune checkpoint inhibitors [7]. A defective entry of CAR T cells in solid tumors after injection was also reported in breast cancer patients [8]. Indeed, upon infusion of patients with T cells expressing a CAR specific for epidermal growth factor receptor 2, engineered CAR T cells were found preferentially in the bone marrow and the liver but not in the solid metastases [8]. To circumvent this defective tumor entry, strategies based on regional delivery of CAR T cells have been put in place with significant success observed even in some aggressive tumors [9,10].

Multiple reasons contribute to this abnormal tumor entry including downregulation of adhesion molecules, or chemokines and the existence of aberrant vasculatures [11]. In addition, infused T cells may not always express the chemokine receptor that binds to the chemokine present in a specific tumor, leading to poor homing. This obstacle can be bypassed by engineering T cells to express a chemokine receptor specific for the chemokine enriched in malignant tissues [12].

Once T cells have extravasated from blood vessels, the second important step that is often overlooked occurs in the tumor stroma (Figure 1). In most solid tumors, the stroma (where blood vessels are located) is a region that surrounds tumor cells, which are organized in nests or islets. This carcinoma structure implies that newly arrived T cells must navigate within the stroma, a very complex microenvironment hostile to the normal functioning of lymphocytes, up to tumor islets. Evidence suggests that this migration step is defective as in many solid tumors the vast majority of resident T cells are located in the stroma and fail to enter the tumor islets where they could get in contact with malignant cells [13]. Remarkably, results of clinical trials have shown that these T-cell-excluded tumors were refractory to PD-1 blockade [14] highlighting the presence of barriers for T cells to infiltrate tumor islets.

Our knowledge of how newly arrived T cells, either conventional or genetically modified, manage to navigate through the stroma and reach cancer cells in a solid tumor is quite limited. This is in part due to the lack of relevant preclinical models that would permit monitoring the dynamic of T cells in the stroma. Of note, most models do not mimic the structure of human carcinomas and are devoid of a stroma.

By using dynamic imaging microscopy on fresh human tumor slices, we have identified several cells and elements contained with the tumor microenvironment (TME) having detrimental impacts on T cells and their ability to reach cancer cells [15,16]. A hallmark of growing tumors is the accumulation of extracellular matrix proteins produced by carcinoma-associated fibroblasts. In non-small cell lung cancers, we demonstrated that thick networks of collagen I fibers, often surrounding tumor islets, prevented T cells from migrating and reaching cancer cells [15]. Since the production of extracellular matrix proteins is performed by fibroblasts, attention has focused on carcinoma-associated fibroblasts (CAF) and their regulation [17]. In this context, TGFβ has emerged as a key factor responsible for the activation of CAF, the excessive production of collagen, and thereby the exclusion of T cells from tumor cell regions [18].

Apart from a physical microenvironment, other cells and determinants have been identified in preventing T cells from entering tumor islets. Our data have highlighted the negative influence of macrophages that by forming long-lived interactions with T cells sequester them in the stroma [16]. Chemokines could also control the positioning of T cells within tumors. Unlike their well-described attractant functions, some chemokines at high doses present some repulsion effects [19]. In pancreatic cancers, the presence of CXCL12 is associated with the lack of T cells in tumor islets by a mechanism that relies on the association of this chemokine with keratin-19 [20,21].

The locomotion of T cells and their proper distribution depends not only on extrinsic factors contained within the TME (such as chemokines, ECM fibers, suppressive cell populations) but also on the intrinsic ability of T cells to migrate and respond to specific chemotactic factors (such as activation and/or exhaustion state, expression of relevant receptors for chemokines or adhesion molecules, metabolic activity to produce the energy required for motility).

As a matter of fact, T cells found in blood and tumors are composed of different subtypes endowed with specific functions. Up to now, efforts have been made to describe their phenotype and link their differentiation status to their antitumor activities [22]. Due to chronic antigen stimulation, a large fraction of T cells in progressing tumors exhibit exhaustion features with reduced functions. However, other T-cell subsets are also present in tumors including lymphocytes with stem cell-like properties. An important notion that emerged these last few years is that these T cells will persist longer than effector lymphocytes and thus will be more potent to control tumor growth [23]. Likewise, the presence of CD8 T memory stem cells (TSCM) in the infusion products will confer a better chance of response to leukemia and lymphoma patients treated with CAR T cells [24,25]. Another T-cell subset of interest for the prospects of T-cell therapy is represented by CD8+ tissue-resident memory T (TRM) [26].

These different T-cell subtypes might harbor different migratory behaviors too, although evidence is scarce at the moment. Notably, all these T-cell subsets express distinct chemokine receptor suggesting that their migration behaviors are different. In this regard, data have been reported showing the presence of specific niches within tumors for several subsets of T cells attracted by specific cues [27,28]. Transcriptomic analysis performed on T cells purified from tumors indicates that TSCM cells express a motility-associated gene expression suggesting active migration [29]. However, that does not necessarily mean that exhausted T cells are defective in their ability to migrate and reach cancer cells. Monitoring migration of endogenous T cells in fresh slices of human tumors, You et al. reported a strong relationship between T-cell motility and the exhausted T-cell state as defined by the expression of CD38 and CTLA-4 [30]. Likewise, in mouse tumors, it was shown that T cells that had entered the tumor within 14 days and that are presumably exhausted migrate faster than T cells that had entered the tumor within four days and that are thus not exhausted [30]. However, many of the markers used to define exhaustion such as PD-1 and CD38 are also expressed by activated T cells. In addition, there is not only one subset of exhausted T cells but several that may harbor different migratory behaviors [31]. The exploration of these different cell types and their respective motility within tumors will be an important area for the future.

Once T cells have finally managed to migrate through the hostile environment of the stroma, they need to engage with tumor cells to perform their effector functions (Figure 1). Results from experiments performed in vitro and confirmed in several organs (e.g., thymus) indicate that antigen recognition triggers T-cell arrest [32]. This stop signal is mediated by TCR-induced signaling pathways including increases in intracellular Ca^2+^ and reorganization of the cytoskeleton [33,34]. Likewise, the activation of the integrins upon TCR engagement stabilizes the interaction between T cells and tumor cells [35]. After a certain amount of time which is highly variable, T cells detach from their targets and regain motility [36]. Over the last years, studies were conducted to compare the behavior of T cells contacting tumor cells through the TCR or through the CAR [37,38]. The notion that emerges from these studies is that CAR T cells, unlike non-modified T cells, do not form stable contact with tumor cells [37]. The reasons are not known but might be due to different signaling triggered by CAR and TCR [39].

## 3. The Role of Immune Checkpoint Proteins in T-Cell Migration within Tumors

Upon T-cell activation that occurs during an immune response, T cells proliferate and produce cytokines, but also upregulate inhibitory surface molecules, including PD-1, that reduce T-cell activation and permit lymphocytes to return to a basal state. It is well established that due to chronic antigen stimulation, tumor-infiltrating lymphocytes (TIL) maintain a high level of PD-1 which contributes to exhaustion. This state of cell dysfunction is characterized by a stepwise and progressive loss of effector functions (including cytotoxicity and proliferation). Exhausted T-cell express multiple inhibitory receptors and show a transcriptional state different from that of functional T cells (for a review, see [40]). The discovery that it was possible to relieve the brake of TIL suppression with immune checkpoint inhibitors (ICI) targeting CTLA4, PD-1, and PD-L1 has revolutionized the treatment of cancer [41]. Although T-cell motility is a determinant factor influencing immunotherapy success, only limited experimental data are available exploring the potential links between immune checkpoints blockade and T-cell migration, especially in a tumoral context.

### 3.1. CTLA-4 and PD-1 Interfere with the Ability of T Cells to Form Stable Conjugates with Their Targets

In vitro co-culture experiments provided the first demonstration that CTLA-4 and PD-1 control T-cell motility and especially the interaction time between T cells and antigen-presenting cells (APC) [42,43,44]. As stated previously, when T cells encounter sufficient activating peptide–MHC complexes they decelerate to form stable conjugates with APC. This stop signal is dependent on the strength of signaling pathways triggered by TCR engagement. Thus, any mechanism able to attenuate TCR-induced signals would in principle reduce the interaction time of T cells with APC or target cells. Accordingly, it was shown that engagement of immune checkpoint proteins with their ligands prevented T cells from forming long-lived interactions with APC cells [42,45,46,47]. Conversely, when the bindings of PD-1 or CTLA-4 with their ligands were blocked, T cells regain stabilized contact with APC [42,47]. Blocking TCR-induced pathways during a normal immune response (e.g., antibacterial or antiviral) has a clear meaning. First, it reduces a prolonged activation of T cells that might be detrimental with a risk of autoimmunity. Second, it enables lymphocytes to disengage from APC and retrieve an active scanning mode.

### 3.2. Blocking CTLA-4 and PD-1 Produces Variable Effects on T-Cell Motility in Tumors

The impact of CTLA-4 and PD-1 on T-cell motility was then evaluated using two-photon microscopy and intravital imaging in mice with tumors or virally infected. In 2012, Mike Dustin’s lab reported the effects of anti-CTLA-4 antibody on tumor growth and on T-cell motility in tumors [48]. It was shown that the treatment of tumor-bearing mice with anti-CTLA-4 antibody failed to induce tumor regression. At the same time, this therapy reduced contact time between T cells and target cells (likely decreasing tumor cell killing), leading indirectly to an increase in intratumoral T-cell motility. However, when combined with radiation therapy, anti-CTLA-4 antibody treatment provoked both tumor regression and T cells to stop on tumor cells (so increasing contact time and favoring tumor cell killing) in an MHC-I and NKG2D-mediated manner, in line with the in vitro data [48]. The notion that ICI restore stabilized conjugates between T cells and tumor cells has recently been confirmed in a mouse melanoma model after the adoptive transfer of cytotoxic T cells. In such a setting, the treatment with anti-PD-L1 and CTLA-4 decreased intratumoral T-cell motility consistent with target engagement [49]. In contrast, Pentcheva-Hoang et al. have reported increased motility of T cells when anti-CTLA-4 antibodies were administered with GVax, a cellular vaccine producing GM-CSF [50]. Similar findings (i.e., an increase in T-cell motility) were reported in the spleen of mice chronically infected with LCMV and treated with anti-PD-1 antibodies [51].

**Figure 2:**
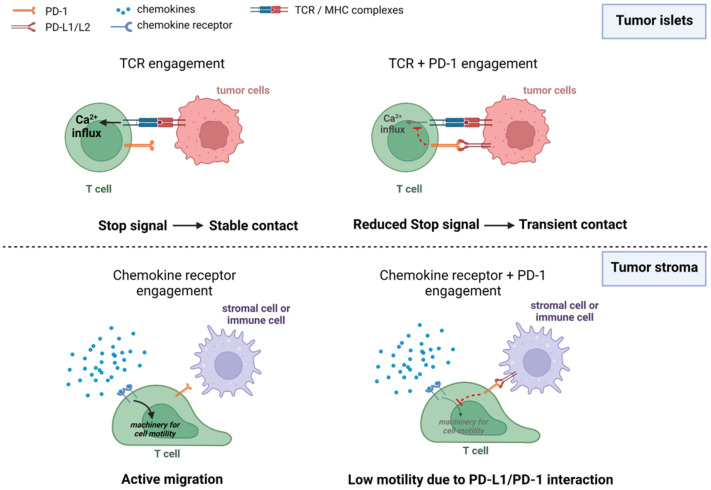
Proposed models of PD-1 engagement on T-cell migration in tumor islets and in the stroma. **Top**: in tumor islets, PD-1 engagement reduces the stop signal and leads to the disengagement of T cells from cancer cells. **Bottom**: in the surrounding stroma, PD-1 engagement reduces T-cell migration by altering signaling pathways triggered by chemokine receptors. Figure created with BioRender.

Several reasons can explain these contradictory findings. First, the motility of T cells in response to ICI may differ depending on their spatial localization (Figure 2). In tumor islets where T cells are in contact with cancer cells, PD-1 and CTLA-4 likely act by interrupting TCR-mediated stop signals. However, this scenario might be very different for T cells located in the stroma. In this compartment, T cells interact with stromal cells including macrophages and dendritic cells which express PD-1 ligands, namely PD-L1 and PD-L2. Yet these cells do not necessarily present tumor antigens to T cells. The impact of PD-1 or CTLA-4 engagement on T-cell motility in the absence of TCR-induced signals is not known for the moment. It is possible that by reducing the signals triggered by chemokine receptors, immune checkpoint proteins mediate inhibitory effects on T-cell migration as opposed to the T-APC disengagement (Figure 2). In such a setting, ICI treatment would increase T-cell trafficking in the stroma and decrease it in the tumor islets.

Second, the duration of the treatment with ICI before measuring the impact on T-cell motility is another important parameter. In this regard, the pro-migratory effects described above were usually observed after several hours-days of treatment with blocking antibodies but not after an acute intervention. In such conditions, it is very difficult to make sure that the enhanced motility is a direct consequence of ICI on T cells. It is known that by relieving the brake of T-cell suppression, fully activated lymphocytes produce more inflammatory cytokines (e.g., IFNγ) known to reprogram the TME, which might be more permissive to T-cell migration. For instance, chemokines such as CXCL9/10, upregulated by IFNγ, are known to attract activated T cells and promote their migration [52,53]. This is consistent with data showing that cancer patients responding to ICI exhibit increased numbers of T cells within tumors [14].

Finally, depending on their differentiation state and level of PD-1 expression, T cells may be affected differently by PD-1 blockade. For instance, discrepancies in the motility of CD4+ and CD8+ T cells following ICI blockade could be explained by their epigenetic profiles [54]. In particular, TOX and NR4A, which are epigenetic regulators of T cells exhaustion and anergy, are also known to impact immune checkpoint expression [55,56]. Interestingly, several studies suggest that PD-1 expression is associated with chronic exposure to antigens due to infections or cancers leading to TOX- and NR4A-dependent immune exhaustion of T cells [54]. However, the implications of PD-1 engagement and blockade of those T cells considered as exhausted on motility are unclear and would depend on the context (acute vs. chronic state for example). For instance, in mice with LMCV persistent infection where T cells are considered exhausted, CD8+ and CD4+ T cells are nearly static in the white pulp. In this setting, PD-1 blockade results in an increase in CD8+ T-cell motility. In addition, transcriptomic analysis performed on human TILs purified from lung tumors indicates that T cells expressing high levels of PD-1 harbor an altered expression of genes involved in cell migration [57].

Another important aspect to consider is the potential response of CAR T cells to ICI with regards to motility and interaction with target cells. Indeed, the studies cited above only focused on analyzing the impact of ICI on the dynamics of endogenous tumor-infiltrating T cells (TILs). However, as previously stated [58], signaling from CAR and TCR receptors differ greatly, and the same could be true for their response to ICI.

First, a suboptimal activation of engineered T cells due to inefficient CAR-mediated signaling compared to TCR-based T cells may impact on CAR T cell motility and their ability to form durable and efficient contacts with target tumor cells. In that respect, CAR T cells have been shown to form a disorganized synapse with tumor cells as opposed to the mature synapse of non-modified T cells [37]. With regards to T-cell motility within tumors, no direct comparison has been made between CAR T cells and TILs.

Second, although encouraging results have been observed for a combined CAR T cell and anti-PD-1 therapy [59,60], the effects of ICI treatments on cell motility have been mainly assessed for endogenous TILs, and their impacts on CAR T-cell motility have still to be determined, especially by comparing them to those observed for endogenous TILs.

Altogether, those studies show the importance of considering the timing, the location, and the type of T cells when interpreting the effects of anti-PD-1 and anti-CTLA-4 blocking antibodies.

## 4. Metabolism and T-Cell Migration within Tumors

### 4.1. A Brief Overview of T-Cell Metabolism

Once matured from the thymus, naïve T cells enter the circulation and start patrolling the tissues looking for specific antigens. At this stage, these cells are considered metabolically quiescent. Indeed, they engage catabolic reactions mainly to sustain cell survival, and the overall levels of both glycolysis and oxidative phosphorylation (OXPHOS) are very low [61]. Upon antigen encounter, T cells undergo a massive metabolic remodeling, greatly increasing anabolism to sustain their clonal expansion [61]. Particularly, T cells can activate a positive feedback loop between signaling pathways and glycolytic enzymes [62], whose expression is greatly enhanced, thus favoring a fast ATP production and the generation of glycolytic intermediates used to sustain nucleotide and lipid synthesis. However, although aerobic glycolysis was long believed to be a hallmark of activated T cells, T cells also display a significant increase in OXPHOS upon activation, although to a lesser extent than glycolysis [63]. Partly, this is required to increase mitochondrial reactive oxygen species (ROS) production, which controls several signaling pathways upon activation [64]. The relative contribution of glycolysis and OXPHOS to T-cell metabolism seems to be intricately interconnected with the differentiation route of activated T cells [65,66]. Indeed, while in effector CD8+ T cells glycolysis is important to promote cytokine expression and effector functions [67], in activated CD8+ T cells primed towards a memory phenotype a relative higher engagement of OXPHOS is associated with the long-term survival of these cells [68]. Particularly, short-chain fatty acids (partly derived from intestine microbiota) have been shown to sustain memory differentiation by increasing OXPHOS through increased glutamine utilization and fatty acid catabolism [69]. Moreover, it has been shown that distinct modes of mitochondrial metabolism support either differentiation (through citrate export and electron transport chain (ETC) complex I) or effector functions (though ETC complex II) in CD4+ T helper1 (T_H_1) cells [70].

### 4.2. Metabolic Determinants Controlling the Motility of T Cells and Comparison with Those of Other Cells

The eukaryotic cytoskeleton is composed mainly of two protein polymers, namely actin and tubulin. These proteins can dynamically polymerize into filaments by consuming energy (ATP for actin, and GTP for tubulin). Such cytoskeletal rearrangements are crucial to sustaining T-cell motility, which therefore is a highly energy-demanding process. ATP can be generated in the cell either through glycolysis (fast rate but poor yield) or OXPHOS (low rate but high yield, although only within mitochondria). GTP can be generated by the enzyme succinyl-CoA-synthetase during the Krebs cycle, or it can be obtained from ATP thanks to the enzyme nucleoside-diphosphate-kinase (NDPK).

The link between metabolic pathways and cell migration has been well studied in a number of cells including cancer cells and spermatozoa. Interestingly, in cancer cells, several glycolytic enzymes have been described to interact with cytoskeletal proteins, such as hexokinase, phosphofructokinase (PFK), and pyruvate kinase (PK) [71]. The latter is one of the two glycolytic enzymes that produce ATP (the other one being the phospho-glycerate kinase). PFK1 (controlling the main rate-limiting step enzyme of glycolysis) can be targeted to degradation upon actin depolymerization [72]. Of note, in cells moving through actin protrusions (such as filopodia and lamellipodia of endothelial cells) at the leading edge, several glycolytic enzymes, such as PK and 6-phosphofructo-2-kinase/fructose-2,6-biphosphatase-3 (PFKFB3), associate with the polymerized actin (F-actin) at the leading edge [73]. Overall, in cancer cells glycolysis seems to be preferred over OXPHOS to sustain a highly dynamic process (i.e., actin remodeling) occurring simultaneously throughout the whole leading edge by providing ATP locally.

By contrast, a different motility is observed in spermatozoa. In the human spermatozoa, mitochondria are enriched in the midpiece of the tail and supply the energy required for the motility together with other factors. The first is the concentration of glucose and fructose in the sperm. While glucose sustains glycolytic ATP production and increases spermatozoid mobility [74], fructose (provided by seminal fluid) appears essential to enhance a vigorous motility enabling spermatozoa to reach the egg for fertilization [75]. Second, the citric acid contained in the sperm positively influences the number and motility of spermatozoa [76]. Third, AMPK (5′-AMP-activated protein kinase), the key energy sensor promoting mitochondrial activity and OXPHOS, is essentially localized in the flagellum and acrosome [77], and it is involved in the regulation of sperm motility and acrosome reaction [78,79]. AMPK is also involved in the management of lipid peroxidation and gamete antioxidant enzymes [78]. In line with this, AMPK inhibition significantly decreases the percentages of motile and rapid spermatozoa [79], while AMPK activators (AICAR, metformin) significantly improved sperm motility [77].

**Figure 3:**
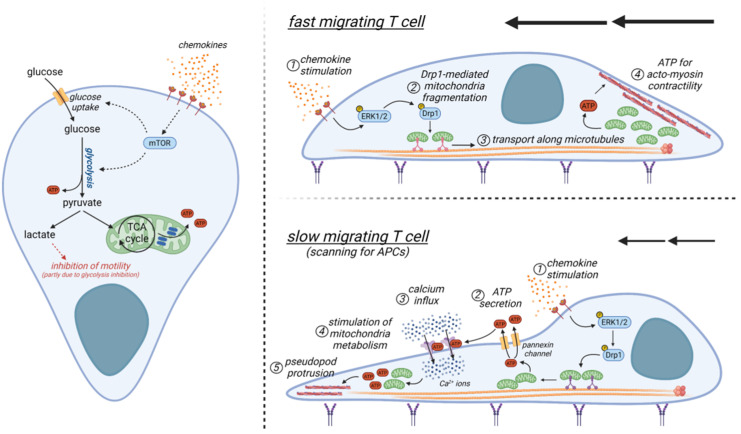
Proposed models of metabolic pathways controlling T-cell migration. **Left**: schematic diagram showing how chemokine receptors can modulate cell metabolism to support ATP production for motility. **Top right**: upon chemokine stimulation, fast migrating T cells translocate their mitochondria towards the cell rear edge in an ERK/Drp1-dependent way. Here mitochondria may produce ATP to sustain actomyosin contractility. **Bottom right**: A feed-forward positive loop between ATP, mitochondria, pannexin (yellow) and purinergic (violet) receptors, and calcium is established in slow migrating T cells to sustain pseudopod formation. Figure created with BioRender.

Contrary to epithelial cell-like and traction-based mesenchymal motility, T cells rely mainly on an ameboid-like migration in tissues, which is less dependent on actin-derived filaments at the leading edge and requires a functional uropod at the rear edge, where the actomyosin motor is located [80]. Here, this motor acts as a propulsor to push the cell forward by generating a retrograde actin flow. Of note, in a 3D environment, T cells can generate such retrograde actin flow without the presence of adhesion molecules, by exploiting only the topographical features of the substrate, thus being autonomous in their locomotive behavior [81]. In addition, an efficient T-cell motility in tissue seems to be proportional to their cortical actomyosin contractility and it can be improved by direct activation of RhoA [82], a key regulator of actomyosin contractility. Remarkably, only a few studies have addressed the metabolic requirements of migrating T cells so far. Glycolysis has been reported to modulate T-cell motility. Indeed, lactate (the end-product of glycolysis) can inhibit CXCL10-driven T-cell motility. Interestingly, extracellular sodium lactate and lactic acid were shown to inhibit the motility of CD4+ and CD8+ T cells, respectively, through different subset-specific transporters which are differentially expressed by CD4+ (Slc5a12) and CD8+ (Slc16a1) T cells [83]. While lactate reduces CXCL10-driven CD4+ T-cell motility by interfering with glycolysis, its effect on CD8+ T-cell motility seems to be glycolysis-independent [83]. In addition, the CCL5 chemokine has been reported to increase glucose uptake, glycolysis, and ATP production in T cells to meet the increased energy demand during cell migration [84], presumably by upregulating mTOR, which is also required to promote the expression of chemotaxis-related proteins. Similarly, stimulation of T cells with either CXCL12 or CCL19/21 chemokine activates ERK1/2 and the mitochondrial pro-fission protein Drp1 [85,86], two molecules known to sustain glycolysis in T cells [86,87]. Moreover, glycolysis promotes cell migration of regulatory T cells, too [88]. In these cells, glycolytic enzyme glucokinase fuels cytoskeletal rearrangements by associating with actin. Apart from glycolysis, OXPHOS seems to play a positive role too in supporting T-cell migration (Figure 3). Mitochondria specifically localize at the uropod during T-cell migration [86,89]. Indeed, chemokines promote the fragmentation of mitochondria through the activation of the mitochondria pro-fission protein Drp1 [86]. Subsequently, fragmented mitochondria can be transported along microtubules towards the cell rear edge [86], where it has been hypothesized that they may act to supply ATP locally to sustain myosin activity [89]. However, during phases of slow locomotion, it has been observed that mitochondria may accumulate also at the leading edge of migrating CD4+ T cells in response to chemokine stimulation [90]. Here, the ATP produced by these organelles is secreted through pannexin-1 channels and then it acts in an autocrine way to stimulate the purinergic P2X4 receptors [90]. This results in a feed-forward signaling mechanism promoting cellular Ca^2+^ influx and sustaining pseudopod protrusion to contact APCs (Figure 3). Of note, during this phase, the metabolism of mitochondria at the cell rear edge seems to be modulated by the local accumulation of P2Y11 receptors, this presumably favoring the production of ATP at the cell leading edge [91].

In sum, both glycolysis and OXPHOS seem to play a role during T-cell migration, although the relative contribution of the two processes is not currently understood. We could postulate that glycolysis and OXPHOS may be alternatively engaged according to the specific locomotion of a T-cell, i.e., slow locomotion (during which the T-cell needs to accurately scan the surrounding environment to find APCs or target cells) or fast motility (required to efficiently infiltrate into tissue) (Figure 3). However, it should be noted that all these studies have been performed using in vitro 2D systems. Therefore, although some of these findings may extend also to in vivo conditions, the actual metabolic requirements of a T-cell in a real 3D environment are completely unknown.

### 4.3. Metabolic Alterations within the Tumor Microenvironment and Their Impact on T-Cell Motility

A solid TME is characterized by different subpopulations of cells in addition to cancer cells, including both pro-tumoral and anti-tumoral immune and stromal cells. Since cancer cells are constantly trying to grow and proliferate, the TME is a tissue greedy for nutrients, which are consumed at high levels by tumoral cells. Consequently, several nutrients required for T cells to proliferate and for their effector functions may not be available in the adequate range, such as glucose, glutamine, tryptophan, and arginine [92]. In parallel, several toxic metabolites can accumulate in the TME as waste products of cancer cell metabolism (such as lactate) or actively produced by immune cells (such as the derivatives of tryptophan metabolism) [93,94]. Although several studies have elucidated how all these alterations may negatively impact T-cell functions (for a review see [95]), either by promoting a pro-tumoral suppressive phenotype or by inhibiting effector functions, we currently ignore if and how these metabolic alterations impact T-cell motility. Based on in vitro data, we can speculate that the reduced availability of glucose and the consequent reduction in glycolytic rates observed in tumor-infiltrating T cells [96] may be associated with reduced motility. In addition, a defective functionality of some glycolytic enzymes in TIL has been reported that may be further responsible for reducing the glycolytic rate, and thus cell motility [97]. Both tryptophan and arginine have been described as positive regulators of cell migration [98,99]. Therefore, their reduced amount in the TME may potentially decrease T-cell migration, although specific studies are lacking. In addition, several waste products accumulating in the TME may inhibit T-cell motility, thus favoring tumor growth and tumorigenicity. For example, lactate can inhibit T-cell motility in vitro [83], and its accumulation in the TME [94] may play a similar role. Finally, the lack of oxygen, a hallmark of growing tumors, can impede the migration of T cells dependent on oxidative phosphorylation. Naive T cells in murine lymph nodes actively rely on a high level of oxygen to migrate [100]. Likewise, in melanoma mouse tumors TIL were shown to concentrate and migrate more actively around blood vessels as compared to avascular areas [101].

T cells within the TME are frequently characterized by the activation of inhibitory co-receptor pathways, such as PD-1, LAG-3, CTLA-4, etc. Several studies have shown that these signaling pathways may negatively regulate T-cell metabolism. For example, PD-1 signaling can reduce both glycolysis and OXPHOS in T cells [102,103]. Indeed, PD-1 engagement on T cell surface during activation can inhibit both the PI3K/Akt/mTOR and MAPK/ERK signaling pathways, both required for an efficient glycolytic engagement in T cells [103,104]. PD-1 has been also reported (*i*) to inhibit the activation of the mitochondrial pro-fission protein Drp1 [85], which is required for T cell motility [86], and (*ii*) to promote disassembly of the mitochondrial *cristae* [102], which are required for OXPHOS-dependent ATP generation. Similarly, CTLA-4 engagement on T cell surface dampens the activation of PI3K/Akt/mTOR [105], and therefore reduces glycolysis. Knock-out of TIM-3 in an in vitro T cell model reduces glucose uptake and glycolysis [106]. Overall, given the connections mentioned above between metabolism and migration in T cells, the effects of these inhibitory co-receptors on T-cell motility described above may also depend on the concomitant alteration of T-cell metabolism. However, no studies have so far addressed this point.

In sum, metabolic alterations in the TME may have the potential to impact T and CAR T cell motility within human solid tumors. Although specific studies addressing these points are still lacking, these concepts should be considered to understand how the TME is able to reduce the effectiveness of CAR T cells and to develop new strategies to improve current immunotherapy approaches based on engineered T cells. It is also worth noting that studying the influence of metabolism on T-cell motility has been performed so far on endogenous T cells. Therefore, the role of metabolism in regulating the dynamic of CAR T-cells is currently ignored, as well as the potential impact of metabolic alterations in the TME on CAR T cell motility.

## 5. Adhesion Molecules Controlling the Contact between CAR T Cells and Their Targets

As stated previously, CAR T cells need to form productive conjugates with their targets via the assembly of an immunological synapse to achieve control of tumor growth [5]. Up to now, little is known about the mechanisms that regulate CAR T-cell interaction with tumor cells. Previous studies performed with non-modified T cells have underlined that, in addition to the recognition of specific peptide-major histocompatibility complex (pMHC) molecules via the T-cell receptor (TCR), engagement of adhesion and costimulatory molecules with their respective ligands is mandatory to trigger efficacious antitumor T-cell activities. Among adhesion/costimulatory molecules, attention has mostly focused on integrins, in particular, lymphocyte function-associated antigen-1 (LFA-1, CD11a/CD18 or αLβ2) and CD103 (αEβ7), which play important roles in T-cell-target cell adhesion through interaction with their respective ligands, intercellular adhesion molecule-1 (ICAM-1 or CD54) and the epithelial cell marker E-cadherin [107,108]. CD2, which binds to CD58 (LFA-3) on target cells, also acts as an adhesion/costimulatory molecule that provides signals to amplify TCR signaling [109].

Although the central role of LFA-1 and CD103 in stabilizing interactions between naturally occurring T cells and tumor target cells is well established, their contribution to CAR T-cell activity is ill-defined. In native T cells, the adhesive properties of integrins are regulated via conformational activation and clustering, initiated by an “inside-out” signaling process emanating at least in part from the TCR [35]. Given the distinct nature of CAR constructs, a key open question pertains to the ability of CAR T cells to properly activate integrins upon tumor cell recognition. This is especially relevant regarding recent studies showing that many aspects of CAR signaling are unique and distinct from endogenous TCR signaling [110]. In particular, the finding that CAR T cells form a disorganized immunological synapse when contacting cancer cells might be a direct consequence of a suboptimal activation of leukocyte integrins [37]. Inefficient integrin activation upon CAR ligation would be particularly critical in conditions of limited expression of adhesion molecules on the surface of tumor cells, in particular ICAM-1, which is frequently downregulated by cancer cells to evade CD8 T-cell mediated destruction [111]. As important support of this model, we have obtained recently published data showing that CAR T efficacy is strongly dependent on the level of tumor cell ICAM-1 [112]. By monitoring intracellular Ca^2+^ responses (a proxy of T-cell activation) of CD20 CAR T cells during their interaction with malignant B cells from chronic lymphocytic leukemia patients, a clear positive correlation was observed between the percentage of activated CAR T cells and ICAM-1 density on tumor cells. A similar process was also observed with EGFR CAR T cells during their contact with carcinoma cells from several cell lines. Under low ICAM-1 expression and despite efficient target antigen expression, EGFR CAR T cells were unable to form productive conjugates with carcinoma cells. Recently, the role of LFA-1-ICAM-1 interaction in CAR T cell activation was confirmed in two independent studies. In the first, the authors exploited the property of the extracellular magnesium (Mg^2+^) to bind to LFA-1 and stabilize its active conformation. Under low Mg^2+^ levels, CAR T-cell activation and cytotoxicity against tumor cells were considerably reduced. Most importantly, in lymphoma patients treated with CD19 CAR T cells, low serum magnesium levels correlated with poor prognosis [113]. The importance of ICAM-1 expression on cancer cells for CAR T-cell activation was revealed in a CRISPR-based screen performed in a multiple myeloma cell line. Invalidation of ICAM-1 gene in tumor cells led to resistance to BCMA CAR T cells [114]. Along the same lines, Majzner’s lab has reported that the loss of CD58 at the surface of tumor cells is associated with CD19 CAR-T cell failure in patients with large B-cell lymphoma (ASH 2020 annual meeting). Thus, the expression of ICAM-1, and possibly other adhesion molecules such as CD58, emerges as a pivotal factor that sets the threshold of CAR T-cell responsiveness.

## 6. Restoring Defective CAR T Cell Migration by Targeting Immune Checkpoint Proteins, Metabolic Factors, and Adhesion Molecules

### 6.1. Promoting CAR T-Cell Interaction with Tumor Cells via Targeting of Adhesion Molecules

#### 6.1.1. Increasing ICAM-1 Expression by Cancer Cells

There are several strategies that can be envisioned to reinforce the interaction between CAR T cells and their targets. Our data indicate that when enough CAR T cells reaches cancer cells, they manage to progressively invade the center of tumor islets. We and others have recently demonstrated the importance of IFNγ produced by activated CAR T cells in this process [112,115]. IFNγ is known to upregulate ICAM-1 expression on tumor cells by triggering a signaling pathway dependent on the phosphorylation of STAT-1. We confirmed this finding and also showed that the pretreatment of cancer cells with IFNγ rendered CAR T cells more efficient in forming conjugates with malignant cells. Hence, increasing IFNγ in the tumor site can be considered a promising strategy to restore CAR T cell-tumor cell interaction through ICAM-1 upregulation. In that respect, T cells expressing CARs with a CD28 costimulatory domain have been shown to release higher quantities of IFNγ than T cells expressing 4-1BB-costimulated CARs [116]. A comparison of the effects of CD28 and 4-1BB costimulatory domains in CAR T- cell activation and interaction with tumor cells will be an important area for the future. However, a high amount of IFNγ can also have a negative influence on CAR T cells due to the increased expression of immunosuppressive molecules like PD-L1 [117]. In addition, not all tumor cells respond to IFNγ due to the presence of molecular aberrations in the IFNγ signaling pathways [118]. Therefore, the possibility to increase ICAM-1 expression in an IFNγ-independent manner is of great interest. In this regard, systemic thermal therapy has been shown to be associated with IL-6 production, leading to ICAM-1 expression by the tumor blood vessels and, thus, favoring the entry of T cells into the tumor site [119]. However, we need to keep in mind the potential negative impact of having ICAM-1 overexpressed in tumor cells. Although required for the formation of a mature synapse, too much ICAM-1 can induce the risk of a prolonged interaction between CAR T cells and their targets leading to overall reduced lymphocyte motility and exhaustion. Up to now, studies with non-modified T cells indicate that this is not the case. Overexpression of ICAM-1 in tumor cells significantly improves T-cell cytotoxicity and effector function, thus leading to tumor regression without apparent negative side effects on T cells [120,121,122]. Further research is needed to know whether the same holds true with engineered T cells.

#### 6.1.2. Increasing Integrin Activity and Expression on CAR T Cells

As reported recently, LFA-1 activation is dependent on the amount of the extracellular Mg^2+^ offering the possibility to exploit this property therapeutically. LFA-1 can also be targeted with a small molecule (7HP349) that activates this integrin. Recent results show that this activator of LFA-1 enhances the immune response triggered by a vaccine against Chagas disease in a mouse model [123].

As stated previously, LFA-1 requires inside-out signals emanating from the TCR or chemokine receptors to be fully activated. Data suggest that CAR ligation is associated with suboptimal integrin activation. Thus, targeting intracellular proteins involved in LFA-1 activation has a good rationale. This includes the protein WNK1 that is activated by TCR signaling and negatively controls the small G protein Rap1 that is necessary for LFA-1 activity. T cells invalidated for WNK1 bind more strongly to ICAM-1 positive endothelial cells [124]. Other negative regulators of LFA-1 represent interesting targets to boost CAR T cell interaction with tumor cells.

### 6.2. Promoting CAR T-Cell Migration in Tumors via Targeting of Metabolism

Considering the importance of oxygen in T-cell migration and the fact that tumors are hypoxic, several strategies have been implemented to overcome this suppressive state. In the first, supplemental oxygen delivery was used in tumor-bearing mice placed in chambers with well-controlled gas composition (60% oxygen). This protocol results in enhanced infiltration of T cells in tumors and improved tumor growth control [125]. Likewise, targeting hypoxia in mouse tumor models with the prodrug TH-302 (Evofosfamide) promotes an influx of T cells into tumor hypoxic zones. Moreover, this treatment combined with ICI provokes an expansion of T cells that were capable of controlling tumor growth [126]. As stated previously, T-cell mitochondrial metabolism plays an important role in T-cell functions. Recently, Amitrano et al. set up a novel optogenetic approach based on light stimulation to specifically and remotely control T-cell OXPHOS. The authors were able to increase mitochondrial efficiency and ATP production in T cells, this having a positive effect on effector functions, including in vitro migration [127]. However, the application of this strategy in cancer immunotherapy requires further studies.

Overall, a better understanding of how T-cell metabolism controls lymphocyte motility holds promise for the development of novel approaches to boost T-cell migration.

### 6.3. Promoting CAR T-Cell Migration in Tumors via Targeting Immune Checkpoint Proteins

Although it is well established that ICI relieve T cells from their exhaustion state, whether they are also capable of promoting T cell migration is not known for the moment and conflicting results have been reported. In our review, we consider that the location, timing, and differentiation state of T cells will dictate the effects of ICI on T cell migration. It is well documented that by dampening CD28 and TCR stop signaling, PD-1 and CTLA-4 ligations enable T cells to disengage with their targets. However, navigating T cells in tumors are also in contact with stromal cells that express PD-L1 and PD-L2. Such cells do not necessarily present antigen to T cells. One of the questions that pertains is whether PD-1 engagement on migrating T cells has any effect on their dynamic behavior. Interestingly, recent evidence indicates it could. Indeed, PD-1 ligation has been shown to interfere with signaling pathways triggered by chemokine receptors through downregulation of ERK and Drp1, a mitochondria pro-fission protein known to favor T cell migration [85]. Clearly, more experiments are needed to confirm this hypothesis. Finally, we believe it is important to consider the role of other ICI on T cell migration such as TIM-3 and LAG-3.

## 7. Conclusions

Identifying resistance mechanisms to CAR T cells is a topic of utmost importance. Possible factors contributing to such resistance have been identified, including inherent CAR T-cell dysfunction, the presence of an immunosuppressive tumor environment, and tumor-intrinsic factors. Currently, many strategies are implemented to increase CAR sensitivity and T-cell persistence. Yet, antitumor activities of engineered T cells also rely on their capacity to migrate and form productive conjugates with cancer cells. To date, several obstacles to T cell locomotion, mostly extrinsic, have been identified. These include a dense extracellular matrix as well as tumor-associated macrophages. In our review, we postulate that the role of other determinants should be considered too, offering novel possibilities to boost a defective intratumoral migration of CAR T cells.

PD-1 blockade has transformed the treatment of multiple malignancies. Whereas it is well established that PD-1 engagement impedes T cell activation, a potential effect of immune checkpoint proteins on the machinery leading to T cell migration cannot be excluded and should be explored.

In recent years, metabolism has become instrumental in controlling T cell activation and differentiation. As a matter of fact, results from clinical trials indicate that the efficacy of the infused T cell products is linked to specific metabolic profiles. Considering the high level of energy required for T cells to move and infiltrate tissues, including tumors, it is reasonable to assume that specific metabolic pathways control the migration of engineered T cells. There is still much to learn about the nature of such pathways and how they can be harnessed to improve T cell migration in tumors.

To form physical contact with cancer cells, T cells have to engage their antigen receptors together with adhesion molecules. Surprisingly, the relevance of adhesion/costimulatory molecules for the efficacy of CAR T cells has received little attention so far. This is probably related to the strong focus most CAR T cells developers have put on optimizing the design of the CAR construct. However, recent reports are putting a new light on determining the role of adhesion molecules besides the CAR. Strategies to increase the expression of adhesion molecules on tumor cells have been proposed as well.

Given the engineered nature of CAR T cells and the possibilities to modify the tumor environment, many opportunities arise to improve CAR T cell migration and the ability to find tumor cells. These strategies represent a dynamic field of research with high potential for clinical applicability.

## Data Availability

Not applicable.
